# Structure of chloramphenicol-bound MexB reveals residues in the distal binding pocket that are critical for substrate recognition

**DOI:** 10.1093/jb/mvag012

**Published:** 2026-02-09

**Authors:** Yushi Ueda, Ryo Yonehara, Etsuko Ishizaka-Ikeda, Atsushi Nakagawa, Eiki Yamashita

**Affiliations:** Institute for Protein Research, The University of Osaka, 3-2 Yamadaoka, Suita, Osaka 565-0871, Japan; Institute for Protein Research, The University of Osaka, 3-2 Yamadaoka, Suita, Osaka 565-0871, Japan; Institute for Protein Research, The University of Osaka, 3-2 Yamadaoka, Suita, Osaka 565-0871, Japan; Institute for Protein Research, The University of Osaka, 3-2 Yamadaoka, Suita, Osaka 565-0871, Japan; Institute for Protein Research, The University of Osaka, 3-2 Yamadaoka, Suita, Osaka 565-0871, Japan

**Keywords:** antibiotics, crystal structure, MexB, multidrug efflux pump, RND transporter

## Abstract

Multidrug resistance in *Pseudomonas aeruginosa* is strongly promoted by the resistance–nodulation–division family tripartite efflux pump MexAB–OprM, whose inner-membrane transporter MexB plays a central role in recognizing and extruding a broad spectrum of antibiotics and detergents. Although crystal structures of MexB have been determined, no structure of MexB bound to an antibiotic has previously been reported. Here, we report crystal structures of drug-free MexB and chloramphenicol-bound MexB crystallized under mildly basic conditions. In the chloramphenicol-bound structure, chloramphenicol binds at the deep end of the distal binding pocket (DBP) groove in the Binding protomer. Based on this structure, we identified DBP residues (Q125, R128, F178, G179, S180 and Q273) that contact chloramphenicol and evaluated their contributions using *in vitro* chloramphenicol resistance assays of single-substitution MexB variants. Substitutions at these positions reduced cell growth in the presence of chloramphenicol, minocycline, levofloxacin and the detergent CYMAL-7. These findings identify a MexB-specific recognition subsite within the DBP groove and provide a structural basis for understanding how MexB recognizes chloramphenicol and other chemically diverse substrates.

## Graphical Abstract

**Graphical Abstract:**
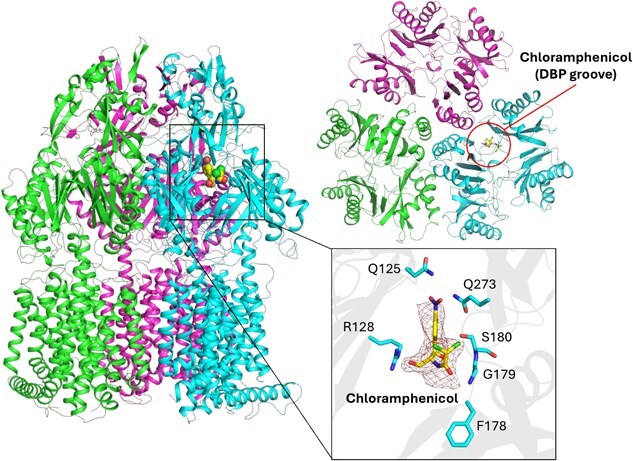

## Abbreviations

ABI-PP: [[2-({[((3R)-1-{8-{[(4-tert-butyl-1,3-thiazol-2-yl)amino]carbonyl}-4-oxo-3-[(E)-2-(1H-tetrazol-5-yl)vinyl]-4H-pyrido[1,2-a]pyrimidin-2-yl}piperidin-3-yl)oxy]carbonyl}amino) ethyl](dimethyl) ammonio] acetate
Chl: chloramphenicol
CYMAL-7: 7-Cyclohexyl-1-Heptyl-β-d-Maltoside
DBP: distal binding pocket
DDM: n-dodecyl-β-d-maltoside
LMNG: Lauryl Maltose Neopentyl Glycol
PBP: proximal binding pocket
RND: Resistance–Nodulation–cell Division

*Pseudomonas aeruginosa* is a representative opportunistic pathogen that exhibits multidrug resistance and causes severe infections, particularly in immunocompromised patients (1). This bacterium has acquired resistance to multiple antimicrobial agents, largely because it possesses tripartite multidrug efflux pumps belonging to the resistance–nodulation–division (RND) superfamily, which form a continuous conduit across the inner and outer membranes and expel antibiotics (2). These tripartite efflux systems consist of an inner-membrane RND transporter that functions as a proton/drug antiporter (3), an outer membrane channel (outer membrane factor, OMF) (4) and a periplasmic adaptor protein (PAP) that bridges the RND transporter and the OMF (5).

Among RND transporters, AcrB from *Escherichia coli* and MexB from *P. aeruginosa* are extensively studied. For these transporters, extensive structural and functional analyses have revealed a common operating mechanism (6, 7). These transporters assemble as trimers, in which the three protomers adopt distinct Access, Binding and Extrusion conformations and cycle cooperatively through these states—the so-called ‘functional rotation mechanism’ that is driven by the proton-motive force (6). Each protomer comprises three domains—the funnel-like (FL) domain, the porter domain and the transmembrane (TM) domain (6). The TM domain consists of 12 helices (TM1–TM12) and harbours residues essential for proton translocation, notably the aspartate residues in TM4 and the lysine residue in TM10. Mutations at these positions abolish coupling to the proton-motive force and render the pump nonfunctional (8). In the Access and Binding states, these aspartate and lysine residues form a salt bridge, whereas in the Extrusion state the lysine instead interacts with a conserved threonine in TM11 (6, 7). The porter domain comprises four subdomains—PN1, PN2, PC1 and PC2. In the Access state, the PN1–PN2 gate is closed and the PC1–PC2 gate is open, permitting substrate uptake from the periplasm. In the Binding state, the PN1–PN2 gate remains closed as in the Access state, whereas the PC1–PC2 gate opens further, thereby configuring an internal binding site. In the Extrusion state, the PN1–PN2 gate opens while the PC1–PC2 gate closes, thereby directing the captured substrate toward the trimer's central channel (6). More recently, in addition to the three canonical states, a ‘resting’ state—where both the PN1–PN2 and PC1–PC2 gates are closed—has been proposed to represent an off-cycle, quiescent conformation of the pump (9, 10). Within the porter domain, two substrate-binding sites—the proximal binding pocket (PBP) (6) and the distal binding pocket (DBP) (11)—are thought to be differentially engaged depending on substrate class and size (12). Substrates reach these pockets via four channels (CH1–CH4) (6, 11, 13–15) that originate in the TM and porter domains, and structural/functional studies indicate that channel usage depends on substrate properties (14, 15).

Among RND efflux systems in *P. aeruginosa*, MexAB–OprM is frequently overexpressed in clinical isolates, where it elevates resistance to multiple unrelated antibiotic classes. In this tripartite system, substrate specificity is determined by the inner-membrane RND transporter MexB, which recognizes and exports chemically diverse compounds, including β-lactams, fluoroquinolones, macrolides, tetracyclines and chloramphenicol (Chl) (1, 2). Despite MexB broad substrate spectrum, structural analyses of MexB complexes have so far resolved only detergent (7, 12, 16) or an inhibitor (16), leaving the structural basis for antibiotic recognition largely unexplored. By contrast, numerous drug-bound AcrB structures have been reported (6, 17, 18), and many of these complexes were crystallized under near-neutral pH conditions (e.g., pH 6.0–6.5). This gap may partly reflect technical constraints: previous MexB crystal structures were predominantly obtained under weakly acidic conditions (e.g., pH 4.5–5.5) (7, 12, 16), which may have hampered systematic efforts to obtain drug-bound complexes. Another factor that may hinder ligand visualization is occupancy of the DBP by purification detergents. In previously reported MexB crystal structures, the DBP is occupied by detergents n-dodecyl-β-d-maltoside (DDM) and lauryl maltose neopentyl glycol (LMNG). To overcome these problems and gain a more comprehensive understanding of how MexB recognizes chemically diverse drugs, we established mildly basic crystallization conditions for MexB using pH 8.0 buffer. This pH condition is closer to physiological conditions than previously reported acidic conditions using pH 4.5–5.5 buffer. Under these conditions, we determined crystal structures of drug-free MexB and Chl-bound MexB. We then identified residues that contribute to substrate recognition and validated their functional significance using *in vitro* drug resistance assays.

## Materials and Methods

### Expression and purification

Transformation, expression and membrane preparation were performed essentially as described previously (19), with minor modifications. Membrane fractions were solubilized by adding 2% (w/v) DDM and incubating the suspension at 4 °C. The solubilized material was clarified by ultracentrifugation at 164,000 × g for 1 h, and the supernatant was mixed with Ni-chelating Sepharose (GE Healthcare) that had been equilibrated with buffer A (50 mM sodium phosphate (pH 7.4), 300 mM NaCl, 20 mM imidazole, 0.02% CYMAL-7). The Ni-resin was washed with buffer B (50 mM sodium phosphate (pH 7.4), 300 mM NaCl, 50 mM imidazole, 0.02% CYMAL-7), followed by an additional wash with buffer C (50 mM sodium phosphate (pH 7.4), 300 mM NaCl, 150 mM imidazole, 0.02% CYMAL-7). MexB was then eluted from the Ni resin with buffer D (50 mM sodium phosphate (pH 7.4), 300 mM NaCl, 350 mM imidazole, 0.02% CYMAL-7). MexB was then eluted from the Ni resin with buffer E (50 mM sodium phosphate (pH 7.4), 300 mM NaCl, 350 mM imidazole, 0.02% CYMAL-7). The eluate was concentrated using a SPIN-X 20 mL centrifugal concentrator (100,000 MWCO; Corning) and subjected to size-exclusion chromatography in two steps. First, the concentrated protein was loaded onto a Superdex 200 16/60 column (Cytiva) equilibrated with buffer F (20 mM sodium phosphate (pH 7.4), 150 mM NaCl, 0.02% CYMAL-7), and peak fractions were collected. These fractions were then applied to a Superdex 200 Increase 10/300 GL column (Cytiva) equilibrated with the same buffer for further purification. Final peak fractions were concentrated to 20 mg/ml and dialyzed against 20 mM sodium phosphate (pH 7.4) containing 0.02% CYMAL-7 prior to crystallization.

### Crystallization

Crystals of MexB were grown at 4 °C by hanging-drop vapor diffusion. Drops containing 1 μl of purified MexB at 20 mg/ml and 1 μl of reservoir solution consisting of 8% (w/v) polyethylene glycol 4000, 0.2 M KCl and 0.1 M HEPES (pH 8.0) were incubated at 4 °C. For cryoprotection, crystals were transferred stepwise through reservoir solutions supplemented with increasing concentrations of glycerol. The glycerol concentration was initially 5% (v/v) and was increased in 5% (v/v) increments every 30 min to a final concentration of 25% (v/v), then the crystals were flash-cooled in liquid nitrogen.

### Soaking of MexB crystals with chloramphenicol

A 0.5-M Chl stock solution was prepared in 100% ethanol. A soaking solution was prepared by supplementing the reservoir solution (0.1 M HEPES (pH 8.0), 0.2 M KCl, 8% (w/v) PEG 4000) with 10 mM Chl. Pre-grown MexB crystals were transferred into the soaking solution and incubated for 24 h at 4 °C.

For cryoprotection of Chl–MexB crystals, the soaking solution surrounding the crystals was stepwise exchanged with soaking solutions containing increasing concentrations of glycerol. The glycerol concentration was initially 5% (v/v) and was increased in 5% (v/v) increments every 30 min to a final concentration of 25% (v/v). The crystals were then harvested and flash-cooled in liquid nitrogen.

### X-ray data collection and structure determination

X-ray diffraction data for MexB were collected at beamline BL44XU (SPring-8, Japan) (20, 21) using MX300-HE detector (Rayonix). Diffraction images were processed with HKL-2000 (22). X-ray diffraction data for Chl were collected using an EIGER X 16 M hybrid photon-counting pixel array detector (Dectris). Diffraction images were processed with autoPROC (23) using XDS (24). Subsequent data handling used Phenix (25). Molecular replacement was carried out with PHASER (26) using the DDM-bound MexB structure (PDB ID 3W9I) (16) as the search model. Model building was performed in Coot (27), and refinement was conducted with Phenix.refine (28), polder maps were generated with phenix.polder (29). Superposition of structures were carried out using the SUPERPOSE in the CCP4 program suite (30). Figures were prepared with PyMOL (Schrödinger). Data collection and structure refinement statistics are summarized in Supplementary Table S7.

### *In vitro* drug resistance assay

Drug resistance assays were performed according to previously reported methods (19, 31), with minor modifications. The *mexA*, *mexB* and *oprM* genes were subcloned into the broad–host–range vector pMMB67HE. Single-amino acid substitution variants were generated by standard PCR-based site-directed mutagenesis, and all primers used for mutagenesis are listed in Supplementary Table S8. The resulting plasmids were transformed into *E. coli* C43(DE3) Δ*acrAB* Δ*tolC* and obtained colonies.

For spot assays, colonies were grown in LB medium containing 100 μg/ml ampicillin for 12 h. Cultures were adjusted to an OD590 of 1.0 in LB. Each adjusted culture medium 4 μl were spotted onto LB agar plates supplemented with 100 μg/ml ampicillin and 0.01 to 2.56 μg/ml Chl. Plates were incubated at 37 °C for 12 h. Control plates lacking Chl were processed in parallel.

Colony growth intensities were quantified using ImageJ (32). For normalization, growth on Chl-free medium for each strain was set to 1, and relative growth on Chl-containing plates was calculated accordingly. Differences between the WT MexB strain and each variant strain were evaluated using two-sided Welch's *t*-tests.

## Results

### Drug-free MexB structure

**Fig. 1:**
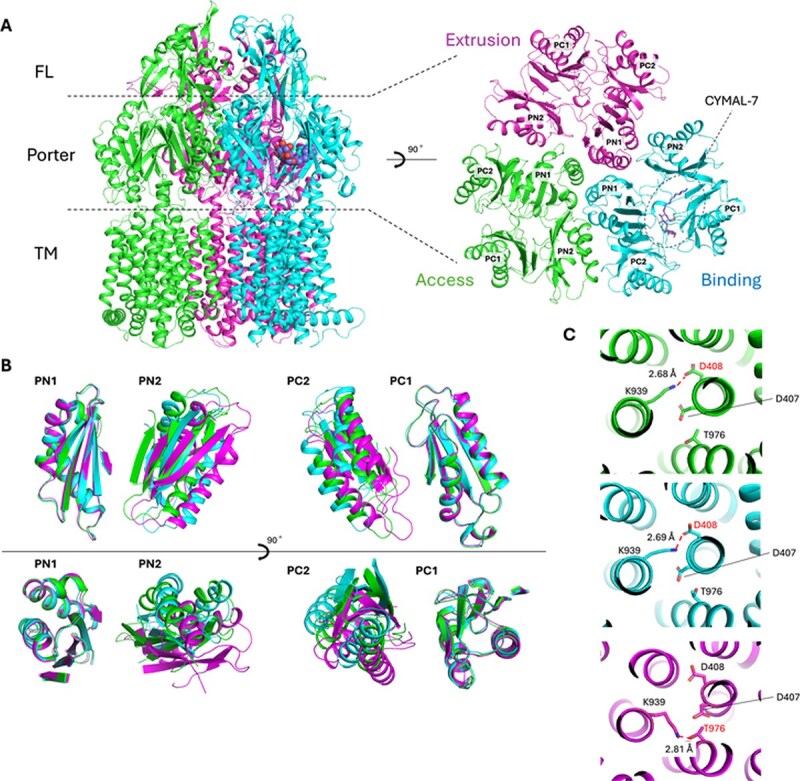
**Structure of drug-free MexB obtained under mildly basic conditions.** (A) Overall structure of the drug-free MexB trimer (left) and top view of the porter domain (right). CYMAL-7 is depicted as spheres or sticks. (B) Superposition of the porter domains from the three protomers. Superpositions were performed using PN1- or PC1-based superpositions. Subdomains are defined as PN1 (40–130, 814–818), PN2 (136–180, 284–328), PC1 (571–666) and PC2 (679–717, 821–858). (C) Comparison of residues implicated in proton translocation across protomers. Side chains of D407, D408, K939 and T976 are shown as sticks. Hydrogen bonds are shown as dashed lines.

We purified *P. aeruginosa* MexB and crystallized it under mildly basic condition, yielding a structure in space group *C*2 at 2.30 Å resolution. The crystallographic asymmetric unit contained one MexB homotrimer. The MexB comprises 1055 residues, of which at least 1024 residues were modelled (Fig. 1A).

Each subunit consists of a TM domain with 12 helices, a porter domain harbouring the substrate-binding pockets and a funnel-like domain. The three protomers are asymmetric and adopt distinct conformations (Fig. 1B and C). The centroid-to-centroid distances between a porter domain subdomains PN1 and PN2 were 26.5, 28.3 and 29.8 Å for chains A, B and C, respectively, and those between a porter domain subdomains PC1 and PC2 were 26.2, 29.1 and 24.5 Å (Supplementary Table S1). Distances among residues involved in proton translocation (MexB D407 and D408 in TM4, K939 in TM10 and T976 in TM11) further distinguished the protomers: in chains A and B, K939–D408 measured 2.68 and 2.69 Å, whereas in chain C, K939–T976 was 2.81 Å (Fig. 1C, Supplementary Table S1). Based on these metrics, we assigned chains A, B and C to the Access, Binding and Extrusion states, respectively. The pairwise Cα root mean square deviations (RMSDs) between our three protomers were 2.34 Å (Access–Binding), 2.59 Å (Access–Extrusion) and 3.34 Å (Binding–Extrusion) (Supplementary Table S2).

Superposition with previously reported MexB structures (16) in the Access, Binding and Extrusion states gave Cα RMSDs of 0.96, 0.66 and 0.74 Å (Supplementary Fig. S1A, Supplementary Table S3), respectively, indicating overall similarity; the Access state showed a slight deviation. In the Access protomer, part of the PC2 Cα4 segment (843–858) together with part of PC2 Cα3 (701–708) tilted toward PC1, while a part of the PC1 Cα2 segment (649–662) shifted outward, away from PC2 (Supplementary Fig. S1A and B). Crystal contacts with a symmetry mate were observed around Cα3, potentially imposing local steric constraints, whereas no symmetry contacts were detected around Cα2 or Cα4. The TM4/TM5 and TM10/TM11 helices likewise exhibited conformational changes. In prior structures (16), TM4, TM5 and TM10 contacted symmetry mates; accordingly, differences in crystal packing likely contribute to the variation in TM domain Cα RMSDs (Supplementary Fig. S2A and B). These domain rearrangements likely account for the elevated Cα RMSD observed for the Access state relative to the reference structure.

**Fig. 2:**
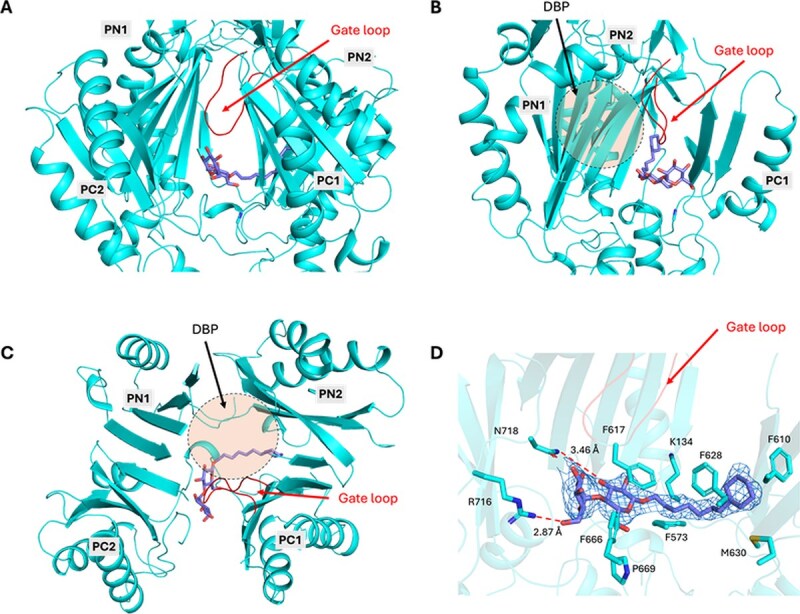
**CYMAL-7 bound to the substrate-binding pocket.** (A) Porter domain of the binding protomer, showing CYMAL-7 positioned adjacent to the gate loop. (B) The view in (A) was rotated 90° about the vertical axis. The foreground PC2 subdomain is omitted to better visualize the binding pocket. (C) Top view of (A) from the periplasmic side, with the view rotated 90° toward the viewer. (D) Close-up view of the CYMAL-7 binding mode. Residues contacting CYMAL-7 are shown as sticks. The Polder map for CYMAL-7 is contoured at 3.0σ and displayed as a mesh. Hydrogen bonds are indicated by dashed lines.

In the Binding protomer, the nonionic detergent 7-cyclohexyl-1-heptyl-β-d-maltoside (CYMAL-7) was located at the entrance between the PC1 and PC2 subdomains (Fig. 2A–C). Its maltoside headgroup was stabilized by hydrophobic contacts with F617, F666 and P669 and by hydrogen bonds with R716 and N718. The hydrophobic moiety primarily contacted residues in PC1 (K134, F573, F610, F628 and M630) and was oriented toward the DBP (Fig. 2D). Thus, CYMAL-7 occupies an intermediate site between the PBPs and DBPs, distinct from the binding positions reported for the purification detergents DDM and LMNG (Supplementary Fig. S3). LMNG has been reported to be transported by the MexAB–OprM system based on functional assays (12). Given that LMNG and DDM occupy overlapping sites (7, 16), DDM may be recognized by MexB. In addition to the density for CYMAL-7 at the PC1–PC2 entrance, we also observed weak positive difference density within the DBP (Supplementary Fig. S4). This density was not sufficiently clear to assign a ligand unambiguously and could arise from crystallization components; accordingly, we did not model any ligand in this site. Separately, substrate resistance assays showed that *E. coli* C43(DE3) *ΔacrAB ΔtolC* expressing wild-type MexB exhibited greater resistance to CYMAL-7 than *E. coli* C43(DE3) *ΔacrAB ΔtolC* harbouring *mexA* and *oprM* but lacking *mexB*, indicating that MexB functionally contributes to CYMAL-7 resistance (Supplementary Fig. S5). Collectively, these data support CYMAL-7 as a substrate of MexB and suggest that the pose captured at the PC1–PC2 entrance represents an early on-pathway intermediate.

### Chloramphenicol-bound MexB

**Fig. 3:**
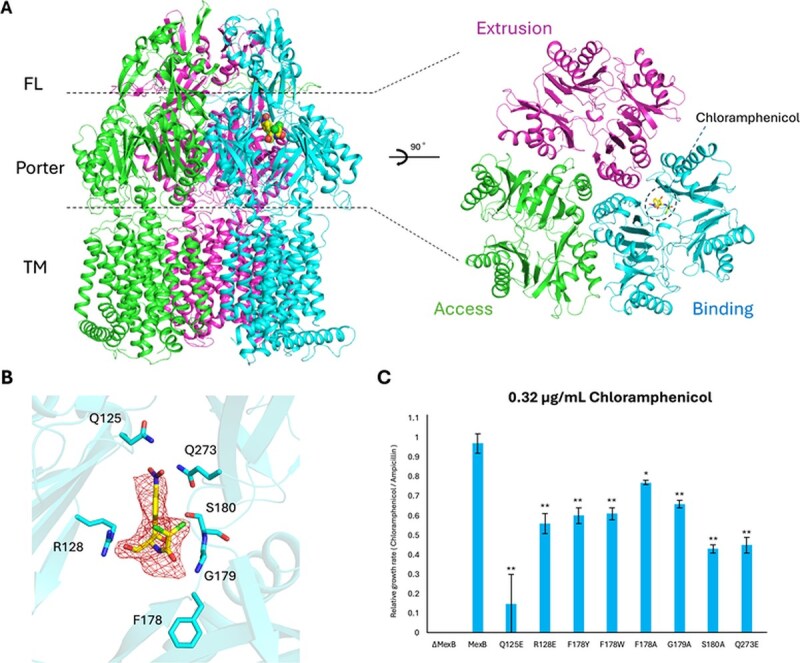
**Structure of the chloramphenicol bound MexB.** (A) Overall structure of the chloramphenicol-bound MexB trimer (left) and top view of the porter domain (right). Chloramphenicol is shown as spheres in the left panel and as sticks in the right panel. (B) Close-up view of the chloramphenicol-binding site. The bound chloramphenicol molecule is shown as yellow sticks, and contacting residues are shown as sticks. The polder map for chloramphenicol is contoured at 3.0 σ and shown as a mesh. (C) Comparison of bacterial growth in the presence of chloramphenicol. *E. coli* harbouring plasmids expressing wild-type MexB or DBP groove mutants were grown on LB agar containing 100 μg/ml ampicillin alone or 100 μg/ml ampicillin with 0.32 μg/ml chloramphenicol. Colony growth was quantified from plate images by measuring colony intensity using ImageJ. Growth ratios were calculated by normalizing the colony intensity on plates containing 100 μg/ml ampicillin with 0.32 μg/ml chloramphenicol to that on plates containing 100 μg/ml ampicillin alone. (^*^P < 0.05, ^**^P < 0.01)

We determined the X-ray crystal structure of MexB in complex with Chl at 2.89 Å resolution. Chl–MexB forms an asymmetric trimer, with protomers adopting the access, binding and extrusion states (Fig. 3A).

Relative to drug-free MexB (space group *C*2 MexB) structure, the Cα RMSDs for the Access, Binding and Extrusion protomers were 0.41 Å, 0.62 Å and 0.46 Å, respectively (Supplementary Table S4). A single Chl molecule was observed in the DBP of the Binding protomer, whereas no Chl density was detected in the Access or Extrusion protomers (Fig. 3A and B). Crystals grown under acidic conditions (pH 4.0) showed no electron density attributable to Chl, whereas crystals grown under mildly basic conditions displayed electron density of Chl. Chl occupied the DBP groove, with no binding observed in the DBP cave or the PBP. Only six residues lay within 3.5 Å of the ligand—Q125, R128, F178, G179, S180 and Q273—comprising polar side chains (Q125, R128, S180 and Q273) together with hydrophobic residues (F178, G179) (Fig. 3B).

In drug-bound AcrB structures, doxycycline (17) and minocycline (18) bind in the DBP groove in a manner like Chl (Supplementary Fig. S6A and B). Two doxycycline molecules are present within the DBP: one bound in the DBP groove and the other in the DBP cave. The doxycycline molecule in the DBP groove binds there through interactions with hydrophobic residues such as F178 and F615, as well as with polar residues including S180, E273 and N274. Minocycline binds at essentially the same position as doxycycline and adopts a very similar pose. By contrast, the aromatic ring of Chl is separated from F178, F615 by > 10 Å, and π–π interaction is not observed in Chl–MexB (Fig. 3B). Levofloxacin is bound within the DBP cave, where it interacts with hydrophobic side chains of AcrB residues such as F178, F610, and F615 (Supplementary Fig. S6C) (17). Because its binding site differs from that of Chl, F178 was the only residue shared between the two ligands in terms of interacting amino acids. In Chl-bound MexB structure, Chl approaches Q125 near the exit gate of the Binding protomer, locating deeper within the DBP groove than previously reported drug-binding sites in AcrB (Supplementary Fig. S6A–C).

In MexB, the detergents DDM and LMNG (7, 12, 16) and the inhibitor ABI-PP (16) occupy the DBP (Supplementary Fig. S7A–D). In those structures, DDM adopts two binding modes: (i) contacting V47, S48, L117, Q125, R128, K151, F178, G179, Q273, D274, I277 and F628 (PDB ID 3W9I) (16); and (ii) contacting V47, S48, Q125, G179, S180, Q273, F615, N616 and F617 (PDB ID 2 V50) (7). The union of DDM contacts therefore includes V47, S48, L117, Q125, R128, K151, F178, G179, S180, Q273, D274, I277, F615, N616, F617 and F628, of which Q125, R128, F178, G179, S180 and Q273 are also engaged by Chl (Supplementary Fig. S7C and D).

LMNG, owing to its larger size, makes extensive contacts across the DBP; residues that overlap with Chl are R128, F178, G179, S180 and Q273, whereas Q125 is not implicated in reported LMNG complexes (Supplementary Fig. S7B). ABI-PP strongly interacts with F178 and binds at a position slightly offset from those of LMNG and DDM. Consequently, residues common to Chl and ABI-PP are limited to R128, F178 and S180, whereas Q125, G179 and Q273 are not shared (Supplementary Fig. S7A).

### *In vitro* drug resistance assay

To validate DBP residues responsible for Chl recognition identified by structure analysis, we prepared single substitution MexB variants (Q125E, R128E, F178Y/W/A, G179A, S180A, Q273E) and assessed susceptibility in Chl supplemented medium.

*Escherichia coli* C43(DE3) Δ*acrAB* Δ*tolC* expressing each MexB variant were cultured in 0.01–2.56 μg/ml Chl. At 1.28 μg/ml Chl, growth of *E. coli* C43(DE3) *ΔacrAB ΔtolC* expressing WT MexB was markedly reduced, and at 2.56 μg/ml Chl, no growth was observed. At concentrations 0.16 μg/ml Chl, *E. coli* C43(DE3) *ΔacrAB ΔtolC* expressing DBP variants and *E. coli* carrying the Δ*mexB* plasmid (vector control) exhibited decreased growth (Table I).

**Table I:** *In vitro* chloramphenicol resistance assay of *E. coli* harbouring inactive pump (∆*mexB*), wildtype MexB and deep binding pocket variants

	**0.01 μg/ml**	**0.02 μg/ml**	**0.04 μg/ml**	**0.08 μg/ml**	**0.16 μg/ml**	**0.32 μg/ml**	**0.64 μg/ml**	**1.28 μg/ml**	**2.56 μg/ml**
**∆MexB**	1.11 ± 0.05	1.08 ± 0.04	1.07 ± 0.04	0.86 ± 0.02 ^*^	0.58 ± 0.19	—	—	—	—
**MexB**	1.10 ± 0.03	1.07 ± 0.03	1.11 ± 0.04	1.04 ± 0.04	1.08 ± 0.06	0.97 ± 0.05	0.73 ± 0.04	0.24 ± 0.14	—
**Q125E**	1.12 ± 0.03	1.08 ± 0.05	1.08 ± 0.02	0.95 ± 0.02	0.78 ± 0.06 ^**^	0.15 ± 0.15 ^**^	—	—	—
**R128E**	1.08 ± 0.03	1.02 ± 0.02	1.00 ± 0.02	0.98 ± 0.07	0.88 ± 0.04 ^*^	0.56 ± 0.05 ^**^	—	—	—
**F178Y**	1.08 ± 0.04	1.00 ± 0.02	0.99 ± 0.02	0.99 ± 0.07	0.89 ± 0.04 ^*^	0.60 ± 0.04 ^**^	—	—	—
**F178W**	1.01 ± 0.03	1.01 ± 0.04	1.01 ± 0.02	0.90 ± 0.03	0.87 ± 0.04 ^*^	0.61 ± 0.03 ^**^	—	—	—
**F178A**	1.02 ± 0.02	0.94 ± 0.03	1.01 ± 0.01	0.93 ± 0.03	0.97 ± 0.03	0.77 ± 0.01 ^*^	0.47 ± 0.06 ^*^	—	—
**G179A**	1.02 ± 0.02	1.01 ± 0.02	1.00 ± 0.02	0.89 ± 0.04	0.85 ± 0.03 ^*^	0.66 ± 0.02 ^**^	—	—	—
**S180A**	1.06 ± 0.02	1.04 ± 0.01	1.01 ± 0.02	0.91 ± 0.08	0.73 ± 0.05 ^**^	0.43 ± 0.02 ^**^	—	—	—
**Q273E**	1.05 ± 0.03	1.03 ± 0.03	1.00 ± 0.02	1.01 ± 0.13	0.78 ± 0.05 ^**^	0.45 ± 0.04 ^**^	—	—	—

Data are shown as mean ± s.e.m. (*N* = 4 biologically independent replicates). *P* values were calculated using a two-tailed Welch's t-test for comparisons between the WT and each mutant. (^*^P < 0.05, ^**^P < 0.01). Cell growth values were normalized to the control (ampicillin). Negative results (pink shading) indicate a reduction in cell growth of ≥15% relative to the control, and darker pink shades correspond to stronger growth inhibition.

At 0.32 μg/ml Chl, *E. coli* C43(DE3) *ΔacrAB ΔtolC* expressing WT MexB showed no detectable decrease in growth. In contrast, all single-substitution DBP variants exhibited reduced growth, with percent decreases in normalized colony intensity (relative to drug-free controls) of 85% (Q125E), 57% (S180A), 55% (Q273E), 44% (R128E) and 23–40% for the remaining variants (Fig. 3C, Table I). Among residues proximal to Chl, substitutions at Q125 produced the largest decrease, followed by S180 and Q273. Substitutions at R128, F178 and G179 had a smaller effect overall.

To assess whether these DBP groove residues also contribute to the recognition of other DBP-binding antibiotics, growth was quantified in the presence of minocycline and levofloxacin, which are known MexB substrates (2, 33, 34) and bind within the DBP of the homologous transporter AcrB (Supplementary Fig. S6B and C). At 0.02 μg/ml minocycline, Q125E, S180A and Q273E showed no detectable growth, whereas R128E showed 83% growth inhibition (Supplementary Table S5). For levofloxacin, at 0.0064 μg/ml, Q125E, S180A and Q273E again showed no detectable growth, while R128E showed 63% inhibition (Supplementary Table S6). Consistent with their strong effects on growth at 0.32 μg/ml Chl (Table I), substitution at Q125, which had the strongest effect on Chl-dependent growth, and substitutions at S180 and Q273, which also substantially reduced growth, likewise impaired resistance to both minocycline and levofloxacin. R128 displayed substrate-dependent effects: modest with Chl (44% inhibition at 0.32 μg/ml), greater with levofloxacin (63% inhibition at 0.0064 μg/ml) and pronounced with minocycline (77% inhibition at 0.02 μg/ml).

The contribution of DBP residues to CYMAL-7 efflux was assessed. *E. coli* C43(DE3) *ΔacrAB ΔtolC* expressing WT MexB grew in the presence of CYMAL-7 at concentrations up to 62.5 μg/ml, whereas the same strain expressing any of the DBP single-substitution variants (Q125E, R128E, F178Y/W/A, G179A, S180A or Q273E) was completely inhibited at 31.2 μg/ml (Supplementary Fig. S5). This result suggests that all DBP variants may be important for CYMAL-7 efflux.

## Discussion

In this study, we determined the crystal structure of Chl-bound MexB by mildly basic crystallization conditions. Chl is positioned at the deep end of the DBP groove (Fig. 3A and B), at a site distinct from those reported for drug-bound AcrB structures (6, 17). The ligand position overlaps with the maltoside headgroups of DDM and LMNG (Supplementary Fig. S7B–D). In the Chl-bound MexB structure determined in this study, we identified residues involved in Chl binding. Based on these structural information, single-substitution DBP variants of MexB (Q125E, R128E, F178Y, F178W, F178A, G179A, S180A and Q273E) were prepared. In the presence of Chl, Q125E markedly decreased cell growth, whereas R128E, S180A and Q273E produced moderate decreases. By contrast, the effects of the F178 variants were small. This pattern is consistent with the structural observation that Chl binding is stabilized by hydrogen bonding rather than π–π stacking. In the presence of minocycline and levofloxacin, Q125E, S180A and Q273E also markedly decreased cell growth, and R128E caused additional reductions with both drugs. In AcrB reference structures, minocycline and levofloxacin bind within the DBP, where they approach residues equivalent to S180 and Q273 but not the Q125-equivalent position (Supplementary Fig. S6A and B). This result suggests that S180 and Q273 in MexB are likely to contribute, at least in part, through direct contacts with DBP-binding antibiotics, whereas Q125, despite not forming obvious contacts in the AcrB complexes, influences efflux from its exit-proximal position. Together with the marked decreases in drug resistance caused by Q125E to Chl, minocycline and levofloxacin, these findings raise the possibility that Q125 acts as an exit-proximal residue that is important for the efflux of substrate, likely interacting with substrates as they progress from the DBP toward the exit gate.

Taken together, these results indicate that Q125, R128, S180 and Q273 are indispensable for MexB-mediated resistance to Chl, minocycline, levofloxacin and CYMAL-7, and that they form a common subsite in the DBP groove that is contacted by chemically diverse substrates. Among these residues, however, the Q125E substitution caused the most severe loss of resistance, identifying this position as the primary residue within the subsite. Consistent with this view, substitutions that introduce an acidic side chain into the same subsite, R128E and Q273E, also reduced MexB-mediated resistance, most likely because the additional negative charges perturb the electrostatic environment in the distal pocket and destabilize ligand binding. A similar result has been reported for AdeB is RND transporter that contributes to multidrug resistance from *Acinetobacter baumannii*. In that study, the N276D mutant, located near the position of MexB residue Q273 in the DBP, decreased resistance to Chl, levofloxacin and minocycline, whereas the E89Q mutant increased resistance to these drugs (17). These observations suggest that modest changes in side-chain charge within this region can affect efflux activity.

The DBP groove of MexB comprises eight hydrophobic and nine polar residues, whereas that of AdeB comprises nine hydrophobic and eight polar residues, indicating that the DBP groove of AdeB is slightly more hydrophobic than that of MexB. In AdeB, the A180S substitution increased resistance to minocycline, whereas in MexB the S180A mutation decreased resistance to this drug, suggesting that recognition of minocycline within the DBP groove is mediated by polar residues. In contrast, the A180S substitution in AdeB and the S180A substitution in MexB both reduced resistance to Chl and levofloxacin. These observations suggest that, in the recognition of Chl and levofloxacin, differences in DBP groove polarity and hydrophobicity may give rise to distinct substrate-binding modes among RND pumps.

We therefore focused on Q125 as the key residue within this MexB subsite and examined how the Q125-equivalent position is configured in other RND transporters (Supplementary Fig. S8). A comparison of primary sequences from 47 transporters revealed that glutamine occupies the Q125-equivalent position in 24 of these 47 RND transporters, whereas glutamate was never observed and aspartate appeared only in MexY and AmrB. When glutamine is not present, this position is most commonly occupied by hydrophobic residues such as leucine (7 of 47) or proline (5 of 47). Thus, glutamine is the predominant residue at the Q125-equivalent position, and acidic residues at this site appear to be extremely rare, indicating that a non-acidic environment is strongly preferred. These observations suggest that, in those RND transporters that retain a glutamine at the position equivalent to MexB Q125, this residue may provide a recognition site for antibiotics and other substrates like that identified in MexB. How alternative residues such as leucine or proline modulate the properties of this site remains unclear and will require future structural and functional analyses.

We have identified a ligand-binding site within the DBP groove of MexB that is used for antibiotic recognition. However, the substrate specificity of MexB is likely to arise from the concerted action of multiple structural elements within this transporter, including the DBP, the PBP, the Gate-loop and the multiple channels. Thus, future structural analyses of MexB in complex with different substrates, in combination with functional studies focusing on the binding sites and transport channels, should provide a more comprehensive understanding of how MexB achieves multidrug recognition.

## Supplementary data

Supplementary Data are available at *JB* Online.

## Supplementary Material

Web_Material_mvag012

## Data Availability

The atomic coordinates have been deposited in Protein Data bank under the accession codes 21FO (drug-free MexB) and 21FP (chloramphenicol-bound MexB).
