# Supplementary material for: Structure of chloramphenicol-bound MexB reveals residues in the distal binding pocket that are critical for substrate recognition
Source: J Biochem. 2026 Feb 9;179(6):403–11. doi: 10.1093/jb/mvag012 (PMC13201268; doi:10.1093/jb/mvag012)
Supplement: Web_Material_mvag012 [file web_material_mvag012.zip › SupplementaryFigure260203.docx]

**Supplementary Figure legends**

**Supplementary Figure 1.** Structural comparison of MexB for each protomer with the previous structure.

(A) Drug-free MexB model shown in ribbon representation, with per-residue Cα RMSD values mapped onto the structure after superposition onto MexB (PDB ID 3W9I).

(B) Superposition of the porter domains of the Access protomers. The previously reported MexB structure (PDB ID 3W9I) is shown in green. In our structure, the PC2 Cα3 and Cα4 segments tilt toward the PC1 side, whereas the PC1 Cα2 segment flares outward.

(C) Superposition of the transmembrane (TM) domains of the Access protomers. The previously reported MexB structure (PDB ID 3W9I) is shown in green.

**Supplementary Figure 2.** Amino acid residues involved in contact with symmetric molecules.

(A) Amino acid residues (pink, stick representation) contacting symmetry molecules in the Access protomer of MexB crystallized under mildly basic conditions.
(B) Amino acid residues (pink, stick representation) contacting symmetry molecules in the Access protomer of MexB in the previously reported structure (PDB ID 3W9I).

**Supplementary Figure 3.** Differences in binding positions between CYMAL-7 and LMNG.

Structural comparison of drug-free MexB (cyan) with LMNG-bound MexB (PDB ID 6IIA, orange), superposed by least-squares fitting of the Cα atoms of residues 1–1030. The foreground PC1 subdomain is hidden to better visualize the detergent-binding site.

**Supplementary Figure 4**. Electron density in the distal binding pocket groove of drug-free MexB.

2Fo–Fc contoured at 1.0 σ (lime green mesh) and Fo–Fc contoured at 3.0 σ (magenta mesh) electron density maps for the DBP groove of drug-free MexB.

**Supplementary Figure 5.** *In vitro* CYMAL-7 resistance of *E. coli* expressing an inactive pump (Δ*mexB*), wild-type MexB, or deep binding pocket variants (Q125E, R128E, F178Y, F178W, F178A, G179A, S180A, Q273E).
CYMAL-7 resistance assays were performed using *E. coli* C43(DE3) Δ*acrAB* Δ*tolC* harboring pMMB67HE-*mexA-mexB-oprM* or *mexB* variants. Cultures were adjusted to an optical density at 590 nm (OD590) of 1.0 and spotted onto LB agar plates containing CYMAL-7 or onto control plates lacking CYMAL-7. All experiments were performed in triplicate.

**Supplementary Figure 6.** Superposition of the chloramphenicol-bound MexB with drug-bound AcrB structures.

(A) Superposition of doxycycline-bound AcrB (PDB ID 7B8R) with chloramphenicol-bound MexB. MexB residues are shown in cyan; AcrB residues are light green.

(B) Superposition of minocycline-bound AcrB (PDB ID 4DX5) with chloramphenicol-bound MexB. MexB residues are shown in cyan; AcrB residues are purple blue.

(C) Superposition of levofloxacin-bound AcrB (PDB ID 7B8T) with chloramphenicol-bound MexB. MexB residues are shown in cyan; AcrB residues are pink.

Corresponding AcrB residues are indicated by colored numbers.

**Supplementary Figure 7.** Structural comparison of chloramphenicol-bound MexB with previously reported MexB structures.
(A) Superposition of chloramphenicol-bound MexB with ABI-PP–bound MexB (PDB ID 3W9J).
(B) Superposition of chloramphenicol-bound MexB with LMNG-bound MexB (PDB ID 6IIA).
(C) Superposition of chloramphenicol-bound MexB with DDM-bound MexB (PDB ID 3W9I).
(D) Superposition of chloramphenicol-bound MexB with DDM-bound MexB (PDB ID 2V50).

**Supplementary Figure 8**. Comparison of the primary sequences of 47 RND transporters.

Residues corresponding to MexB positions 125, 128, 180, and 273 are indicated by red, blue, green, and orange arrows, respectively.

**Supplementary Figures**


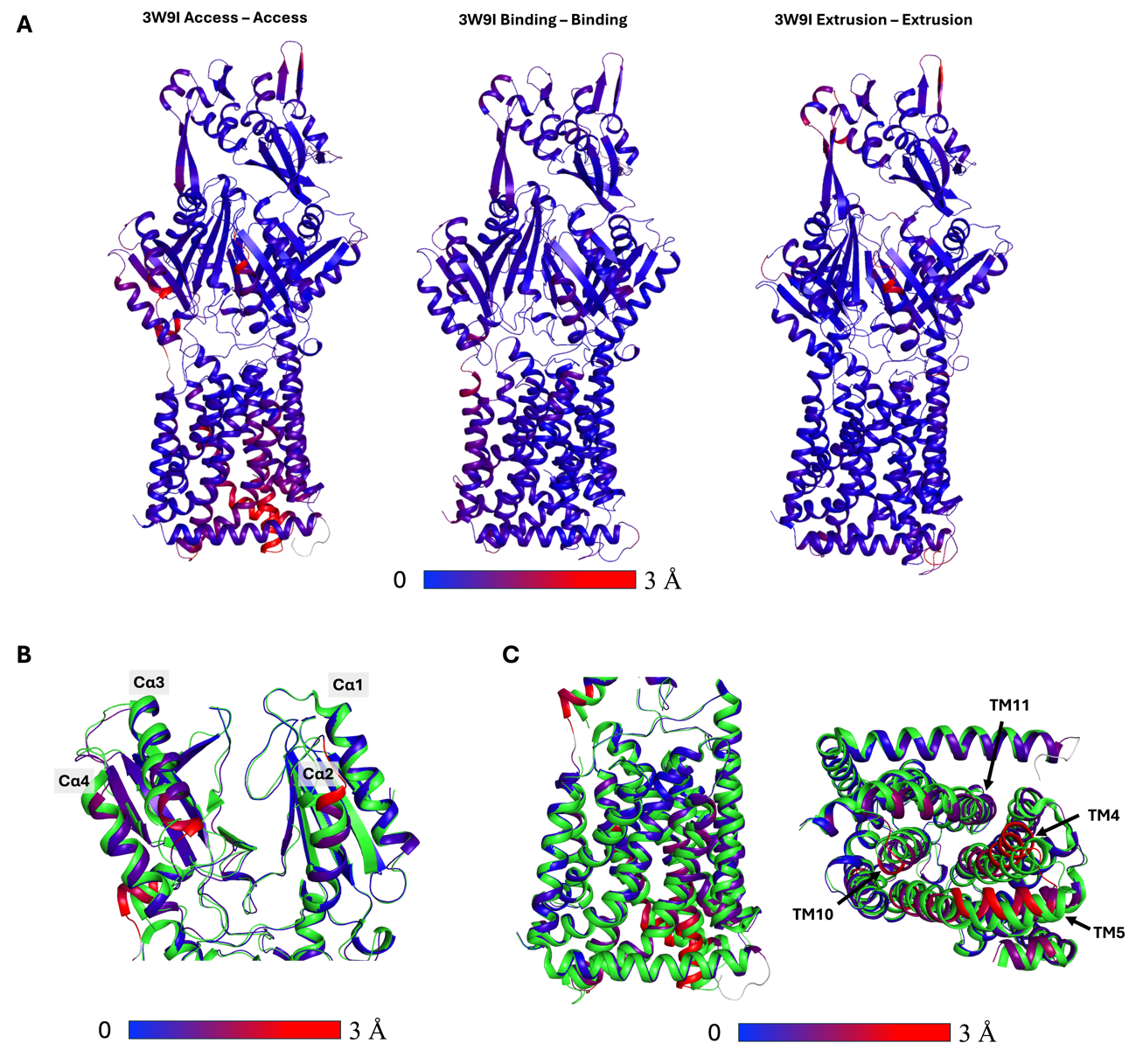


Supplementary Figure 1


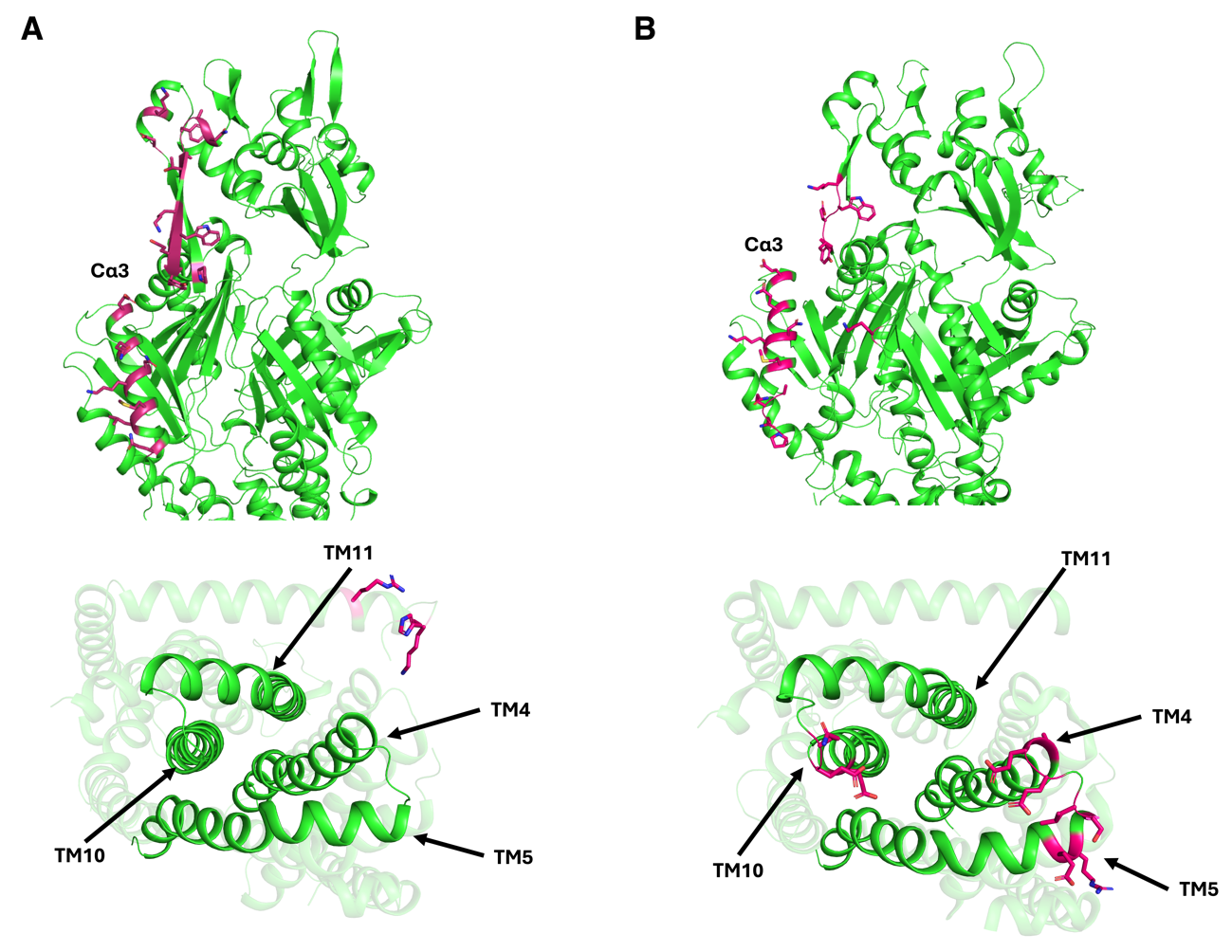


Supplementary Figure 2


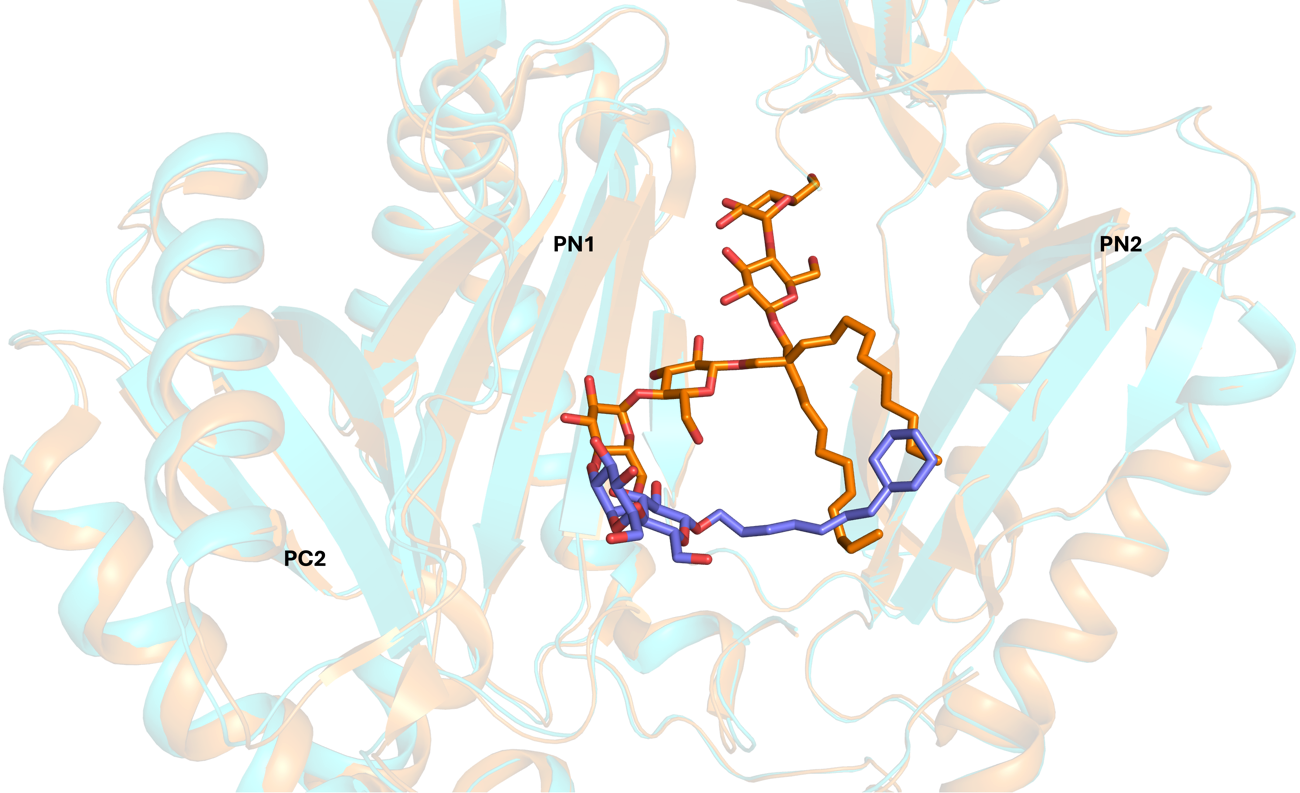


Supplementary Figure 3


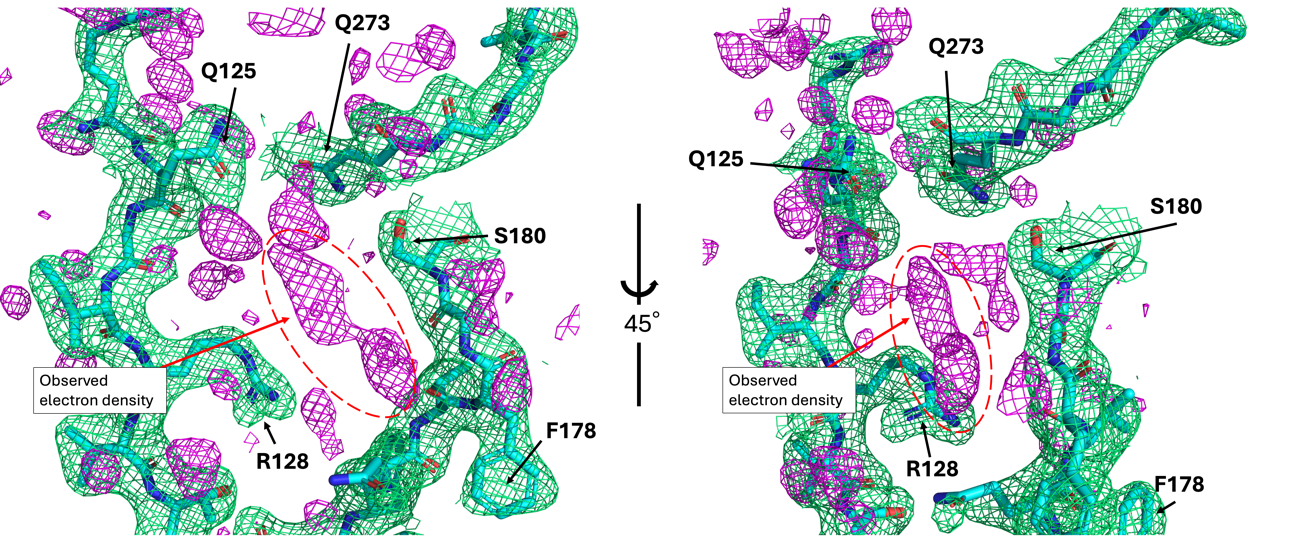


Supplementary Figure 4


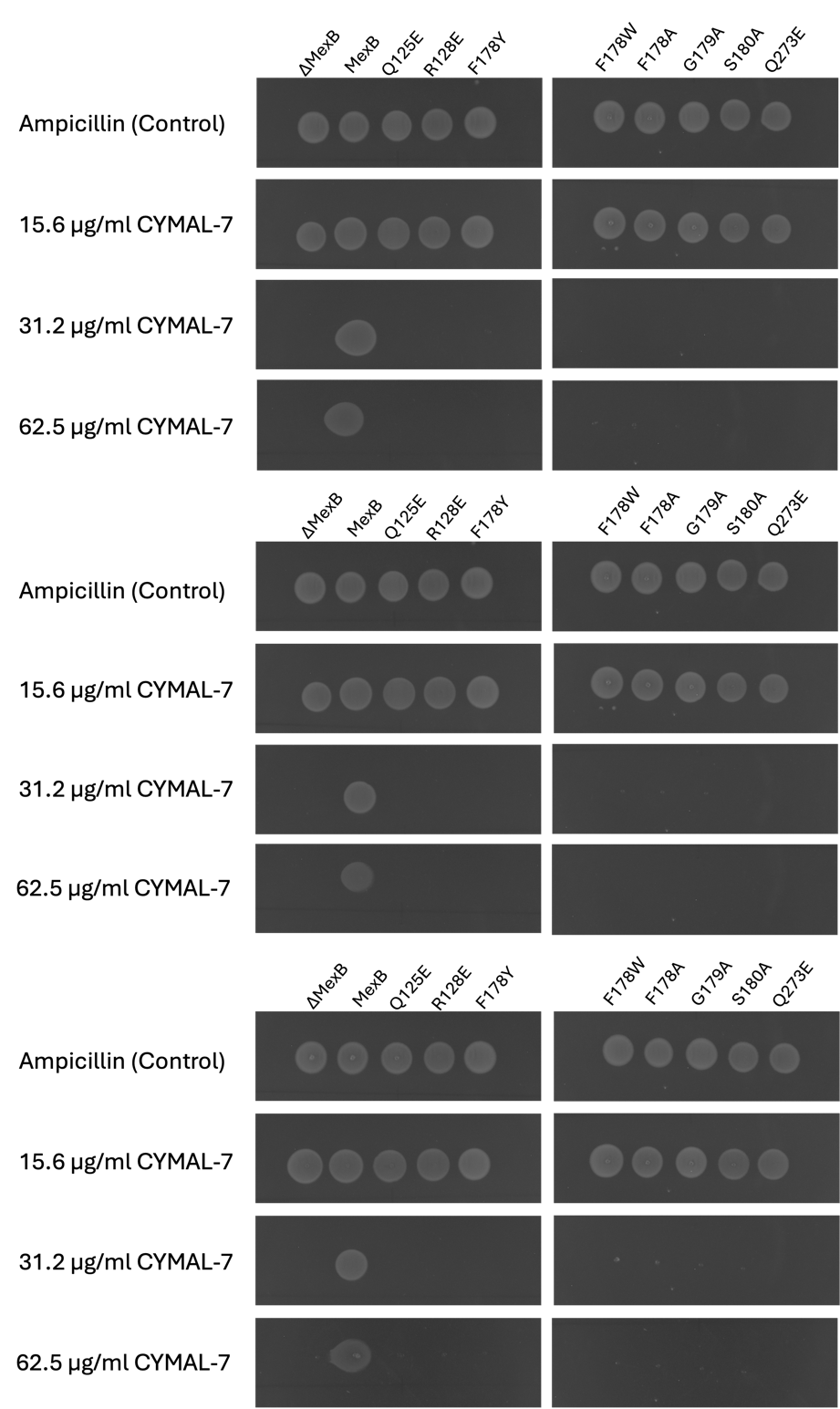


Supplementary Figure 5


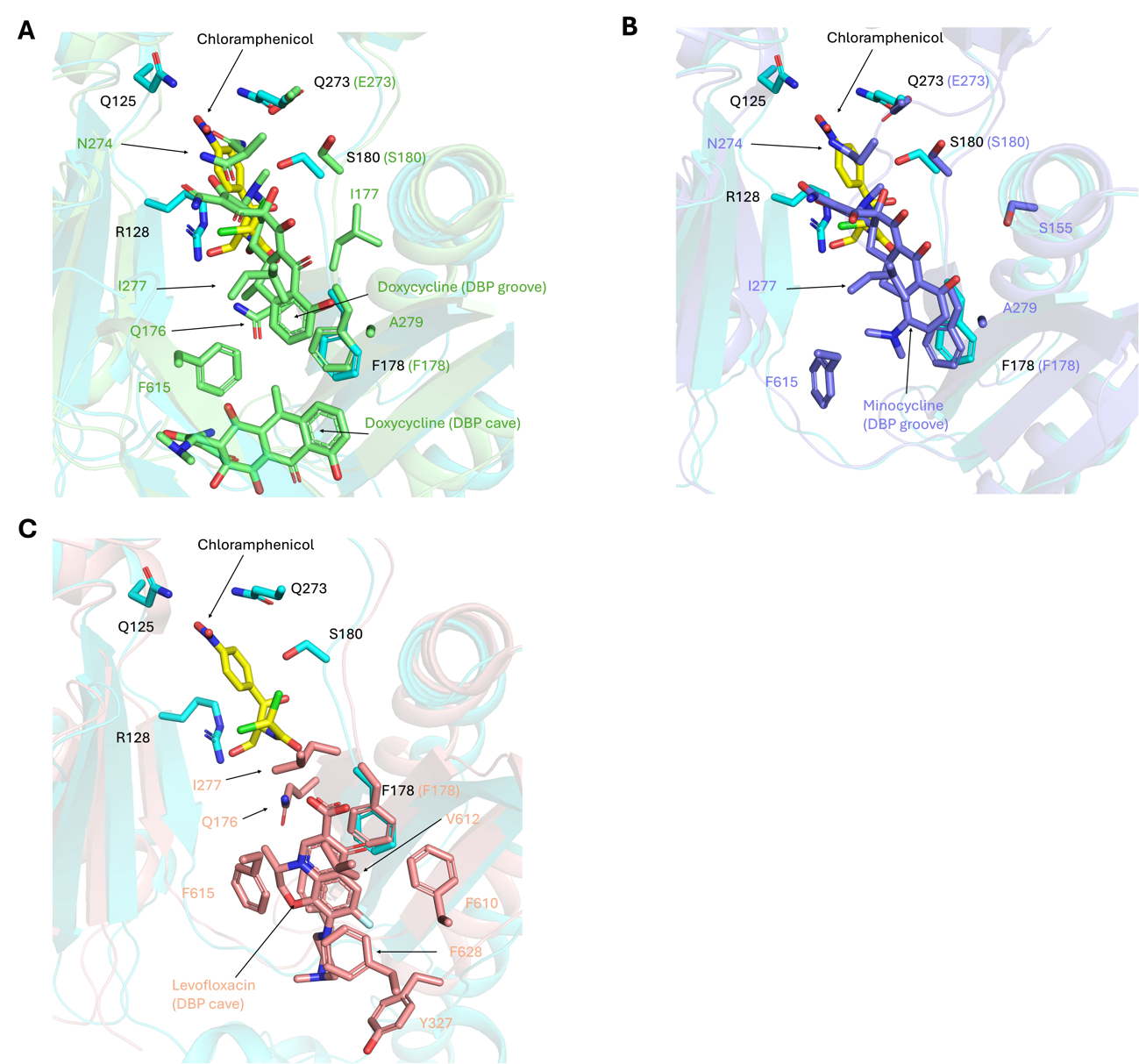


Supplementary Figure 6


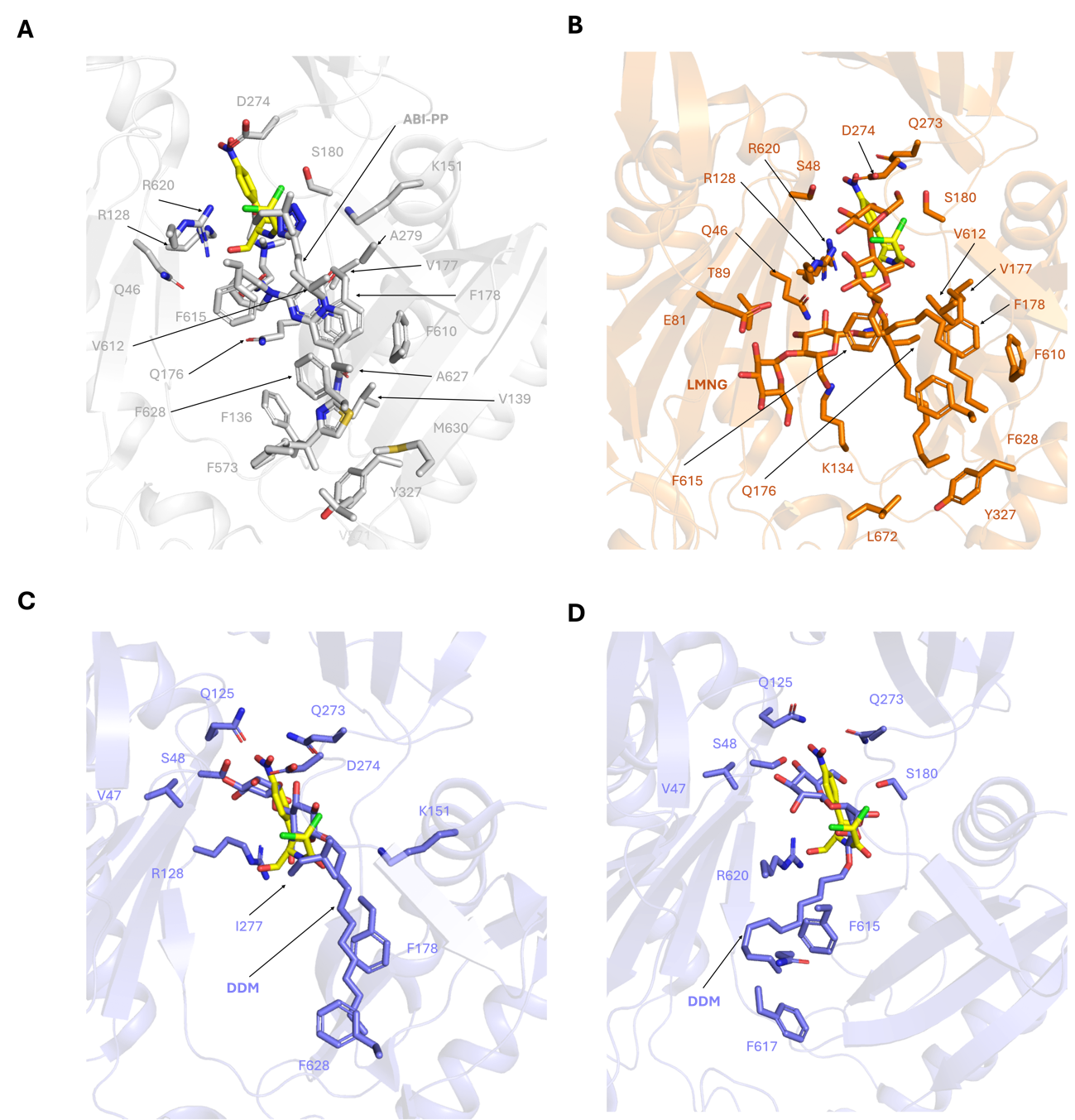


Supplementary Figure 7


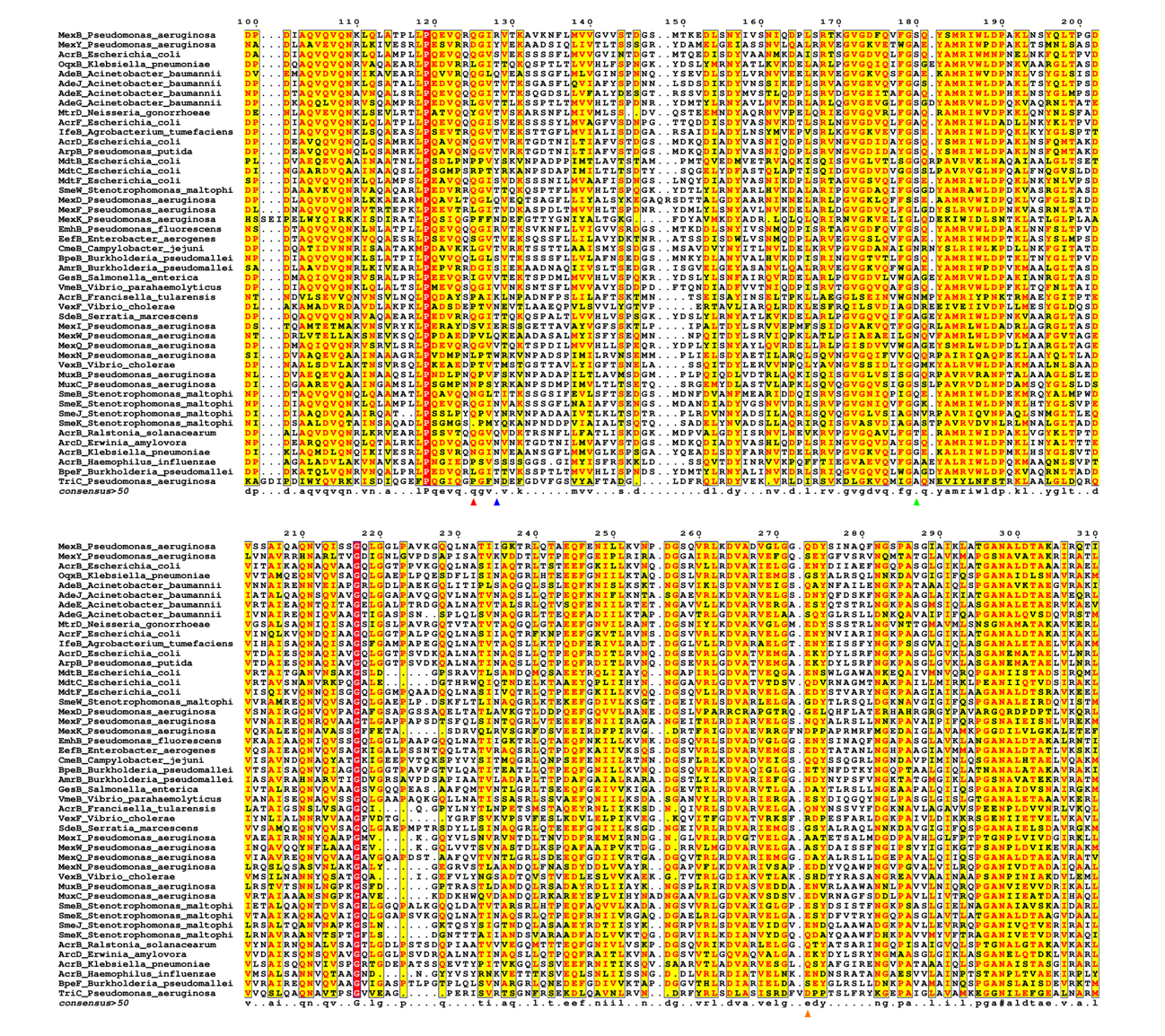


Supplementary Figure 8
