# Supplementary material for: Structure of chloramphenicol-bound MexB reveals residues in the distal binding pocket that are critical for substrate recognition
Source: J Biochem. 2026 Feb 9;179(6):403–11. doi: 10.1093/jb/mvag012 (PMC13201268; doi:10.1093/jb/mvag012)
Supplement: Web_Material_mvag012 [file web_material_mvag012.zip › SupplementaryTable_260128.docx]

**Supplementary Table**

**Supplementary Table Ⅰ.** Center-of-mass distances between subdomains within the porter domain and distances between residues involved in proton transfer in each chain.

|  | **Distance (Å)** | | |  |
| --- | --- | --- | --- | --- |
|  | **PN1-PN2 ^a^** | **PC1-PC2 ^b^** | **D408-K939** | **K939-T976** |
| **Chain A** | 26.5 | 26.2 | 2.68 | 8.41 |
| **Chain B** | 28.3 | 29.1 | 2.69 | 6.78 |
| **Chain C** | 29.8 | 24.5 | 9.04 | 2.81 |

^a^ PN1 (40–130 and 814–818), and PN2 (136–180 and 284–328)

^b^ PC1 (278–281 and 571–666), and PC2 (679–717 and 821–858)

**Supplementary Table Ⅱ.** Cα atom RMSD of Drug-free MexB each protomer.

|  | **Chain A** | **Chain B** | **Chain C** |
| --- | --- | --- | --- |
| **Chain A** |  | 2.34 | 2.59 |
| **Chain B** |  |  | 3.34 |
| **Chain C** |  |  |  |

The Cα RMSD values (Å) for residues 1–1025 in each protomer were calculated using the program SUPERPOSE in the CCP4 suite.

**Supplementary Table Ⅲ.** Cα atom RMSD of each protomer relative to reported MexB.

|  |  | MexB (PDB ID 3W9I) | | | | | |
| --- | --- | --- | --- | --- | --- | --- | --- |
|  |  | **Chain A** | **Chain B** | **Chain C** | **Chain D** | **Chain E** | **Chain F** |
| Drug-free  MexB | **Chain A** | 0.96 | 2.43 | 3.32 | 0.95 | 2.46 | 2.65 |
|  | **Chain B** | 2.60 | 0.66 | 3.32 | 2.70 | 0.70 | 3.37 |
|  | **Chain C** | 2.85 | 3.32 | 0.74 | 2.76 | 3.37 | 0.72 |

The Cα RMSD values (Å) for residues 1–1025 in each chain were calculated using the program SUPERPOSE in the CCP4 suite.

**Supplementary Table Ⅳ.** Comparison of chloramphenicol-bound and drug-free MexB protomers.

|  | **Chain A** | **Chain B** | **Chain C** |
| --- | --- | --- | --- |
| **Chain A** | 0.41 | 2.44 | 2.62 |
| **Chain B** | 2.33 | 0.62 | 3.38 |
| **Chain C** | 2.60 | 3.39 | 0.46 |

The Cα RMSD values (Å) for residues 1–1025 in each chain were calculated using the program SUPERPOSE in the CCP4 suite.

**Supplementary Table Ⅴ.** *In vitro* minocycline resistance assay of *E. coli* harboring inactive pump (∆*mexB*), wildtype MexB, and deep binding pocket variants.

|  | **0.005 µg/mL** | **0.01 µg/mL** | **0.02 µg/mL** | **0.04 µg/mL** | **0.08 µg/mL** | **0.16 µg/mL** |
| --- | --- | --- | --- | --- | --- | --- |
| **∆MexB** | 1.08 ± 0.12 | 0.68 ± 0.01 | - | - | - | - |
| **MexB** | 1.09 ± 0.13 | 0.99 ± 0.13 | 0.78 ± 0.09 | 0.69 ± 0.13 | 0.51 ± 0.18 | - |
| **Q125E** | 1.05 ± 0.08 | 0.86 ± 0.05 | - | - | - | - |
| **R128E** | 1.08 ± 0.14 | 0.91 ± 0.08 | 0.17 ± 0.17 | - | - | - |
| **F178Y** | 1.09 ± 0.09 | 0.90 ± 0.08 | 0.63 ± 0.04 | - | - | - |
| **F178W** | 1.09 ± 0.09 | 0.82 ± 0.07 | 0.68 ± 0.06 | - | - | - |
| **F178A** | 1.09 ± 0.10 | 0.97 ± 0.03 | 0.78 ± 0.07 | 0.16 ± 0.16 | - | - |
| **G179A** | 1.09 ± 0.11 | 0.81 ± 0.07 | 0.69 ± 0.08 | - | - | - |
| **S180A** | 1.02 ± 0.08 | 0.82 ± 0.03 | - | - | - | - |
| **Q273E** | 0.99 ± 0.04 | 0.85 ± 0.04 | - | - | - | - |

Data are shown as mean ± s.e.m. (N = 3 biologically independent replicates). Cell growth values were normalized to the control (ampicillin). Negative results (pink shading) indicate a reduction in cell growth of ≥15% relative to the control, and darker pink shades correspond to stronger growth inhibition.

**Supplementary Table Ⅵ.** *In vitro* levofloxacin resistance assay of *E. coli* harboring inactive pump (∆*mexB*), wildtype MexB, and deep binding pocket variants.

|  | **0.0001 µg/mL** | **0.0002 µg/mL** | **0.0004 µg/mL** | **0.0008 µg/mL** | **0.0016 µg/mL** | **0.0032 µg/mL** | **0.0064 µg/mL** | **0.0128 µg/mL** |
| --- | --- | --- | --- | --- | --- | --- | --- | --- |
| **∆MexB** | 1.00 ± 0.04 | 1.06 ± 0.09 | 0.96 ± 0.02 | 0.99 ± 0.04 | 1.03 ± 0.11 | 0.95 ± 0.07 | - | - |
| **MexB** | 0.99 ± 0.04 | 1.07 ± 0.11 | 1.01 ± 0.04 | 1.00 ± 0.03 | 1.04 ± 0.13 | 1.00 ± 0.10 | 0.71 ± 0.05 | - |
| **Q125E** | 1.00 ± 0.02 | 1.11 ± 0.11 | 1.03 ± 0.04 | 1.04 ± 0.04 | 1.03 ± 0.11 | 0.88 ± 0.06 | - | - |
| **R128E** | 1.01 ± 0.06 | 1.09 ± 0.12 | 1.01 ± 0.06 | 1.01 ± 0.06 | 1.05 ± 0.14 | 1.01 ± 0.13 | 0.37 ± 0.21 | - |
| **F178Y** | 1.02 ± 0.04 | 1.11 ± 0.10 | 1.06 ± 0.09 | 0.99 ± 0.04 | 1.03 ± 0.12 | 1.00 ± 0.10 | 0.64 ± 0.08 | - |
| **F178W** | 1.01 ± 0.02 | 1.15 ± 0.10 | 1.14 ± 0.08 | 1.02 ± 0.03 | 1.01 ± 0.11 | 1.01 ± 0.09 | 0.51 ± 0.18 | - |
| **F178A** | 1.00 ± 0.03 | 1.10 ± 0.11 | 1.15 ± 0.08 | 1.02 ± 0.04 | 1.05 ± 0.14 | 1.02 ± 0.11 | 0.75 ± 0.05 | - |
| **G179A** | 1.07 ± 0.08 | 1.15 ± 0.11 | 1.03 ± 0.03 | 1.02 ± 0.04 | 1.06 ± 0.13 | 0.94 ± 0.14 | 0.74 ± 0.11 | - |
| **S180A** | 0.97 ± 0.04 | 1.18 ± 0.16 | 0.99 ± 0.05 | 0.98 ± 0.06 | 0.98 ± 0.09 | 0.88 ± 0.07 | - | - |
| **Q273E** | 0.95 ± 0.03 | 1.11 ± 0.12 | 0.95 ± 0.02 | 0.95 ± 0.02 | 0.96 ± 0.08 | 0.87 ± 0.05 | - | - |

Data are shown as mean ± s.e.m. (N = 4 biologically independent replicates). Cell growth values were normalized to the control (ampicillin). Negative results (pink shading) indicate a reduction in cell growth of ≥15% relative to the control, and darker pink shades correspond to stronger growth inhibition.

**Supplementary Table Ⅶ.** Crystallographic data and refinement statistics.

|  | **Drug-free MexB** | **Chloramphenicol-bound MexB** |
| --- | --- | --- |
| **Data collection** |  |  |
| Wavelength (Å) | 0.9000 | 0.9000 |
| Space group | *C*2 | *C*2 |
| Cell dimentions |  |  |
| *a*, *b*, *c* (Å) | 207.0, 168.9, 172.2 | 204.9, 169.2, 171.9 |
| *α*, *β*, *γ* (°) | 90.0, 102.2, 90.0 | 90.0, 102.2, 90.0 |
| Resolution (Å) | 40.55-2.30 (2.34-2.30) | 51.02-2.89 (2.94-2.89) |
| Unique reflections | 251734 | 126964 |
| *R*_merge_ | 0.080 (>1.0) | 0.111 (>1.0) |
| Redundancy | 4.0 (4.0) | 7.4 (6.8) |
| CC1/2 | 0.999 (0.406) | 0.991 (0.353) |
| Completeness (%) | 99.5 (100) | 100 (100) |
| *I*/σ (*I*) | 24.1(1.0) | 10.3 (0.6) |
| **Refinement** |  |  |
| Resolution (Å) | 40.55-2.30 | 51.02-2.89 |
| *R*work / *R*free | 0.2204/0.2552 | 0.2237/0.2798 |
| R.m.s.d. from ideal geometry |  |  |
| Bond lengths (Å) | 0.008 | 0.009 |
| Bond angles (°) | 0.99 | 1.17 |
| Ramachandran plot |  |  |
| Favored (%) | 97.89 | 92.39 |
| Allowed (%) | 2.11 | 7.57 |
| Outliers (%) | 0.00 | 0.03 |

Values in parentheses are for the highest resolution shell.

**Supplementary Table Ⅷ.** Lists of primers used in this study.

| **Primer name** | **Sequences (5’-3’)** |
| --- | --- |
| MexB_Q125E_Forward | GAAGTGCAGCGCGAAGGGATCCGGGTG |
| MexB_Q125E_Reverse | CACCCGGATCCCTTCGCGCTGCACTTC |
| MexB_R128E_Forward | CAGCGCCAGGGGATCGAAGTGACCAAGGCGGTG |
| MexB_R128E_Reverse | CACCGCCTTGGTCACTTCGATCCCCTGGCGCTG |
| MexB_F178Y_Forward | GGCGTCGGTGACTATCAGGTGTTCGGC |
| MexB_F178Y_Reverse | GCCGAACACCTGATAGTCACCGACGCC |
| MexB_F178W_Forward | GGCGTCGGTGACTGGCAGGTGTTC |
| MexB_F178W_Reverse | GAACACCTGCCAGTCACCGACGCC |
| MexB_F178A_Forward | GTGACTTCCAGGTGGCCGGCTCGCAGTAC |
| MexB_F178A_Reverse | GTACTGCGAGCCGGCCACCTGGAAGTCAC |
| MexB_G179A_Forward | TTCCAGGTGTTCGCATCGCAGTACTCG |
| MexB_G179A_Reverse | CGAGTACTGCGATGCGAACACCTGGAA |
| MexB_S180A_Forward | CAGGTGTTCGGCGCACAGTACTCGATG |
| MexB_S180A_Reverse | CATCGAGTACTGTGCGCCGAACACCTG |
| MexB_Q273E_Forward | GTAGGCCTGGGCGGCGAAGACTACAGCATCAAC |
| MexB_Q273E_Reverse | GTTGATGCTGTAGTCTTCGCCGCCCAGGCCTAC |
